# Assessing Willingness to Pay for Genetic Testing Among Adults: A Cross-Sectional Study Using Data from the Omnibus Survey 2022

**DOI:** 10.3390/jpm16030154

**Published:** 2026-03-07

**Authors:** Angelo Navas, Lauren Hendy, Megan Roberts

**Affiliations:** Division of Pharmaceutical Outcomes and Policy, Eshelman School of Pharmacy, University of North Carolina, Chapel Hill, NC 27599, USAmegan.roberts@unc.edu (M.R.)

**Keywords:** population genetic screening, willingness to pay, health insurance, beneficiary inducement statue

## Abstract

**Background:** Population genetic screening (PGS) serves an essential role in identifying individuals at higher risk for hereditary cancer and cardiovascular disease. Nevertheless, the current lack of insurance coverage for screening costs might pose a barrier to its adoption. Health systems might contemplate covering these test expenses, but individuals covered by Medicaid and Medicare may not qualify for cost-free screening due to constraints related to the Beneficiary Inducement Statute. **Methods:** A cross-sectional online survey was administered to 602 US adults in January 2023. Andersen’s model guided variable selection. An ordered probit model was deployed to explore the association between insurance type and willingness to pay (WTP) for PGS, controlling for demographic and healthcare characteristics. **Results:** Among the 602 respondents, 524 (87%) were included in our analysis. Over 70% (n = 373) of participants expressed WTP for genetic testing. A similar proportion of respondents with Medicare and Medicaid expressed WTP for screening (68%, and 70%, respectively). Insurance type was not significantly associated with WTP for genetic testing. Notably, lower trust levels and absence of family cancer history were associated with a lower probability of expressing high WTP compared to the reference categories (high levels of trust and having a family cancer history). **Conclusions:** WTP for genetic testing was not significantly associated with insurance type. Almost 30% of our sample were unwilling to pay for PGS, suggesting variability in WTP for PGS and adding to the limited literature on how individuals value genomic screening tests.

## 1. Introduction

Population genetic screening (PGS) plays a crucial role in identifying individuals at a higher risk of developing certain types of cancer and cardiovascular disease, enabling early detection and intervention [1,2,3,4]. The identification of specific genetic markers associated with increased cancer and cardiovascular risks can guide healthcare professionals to deliver more targeted screening strategies, leading to improved health outcomes [5,6]. Over the past decade, the potential for genetic screening among the wider population has attracted more attention [3]. The reason for this increasing interest is because of improved sequencing abilities, reduced testing expenses, and a deeper understanding of the clinical significance and usefulness of gene–disease associations [7,8].

It is estimated that more than 1% of the population carries a pathogenic variant associated with hereditary breast and ovarian cancer syndrome, Lynch syndrome, or familial hypercholesterolemia [9]. These three conditions are designated by the Centers for Disease Control and Prevention (CDC) as Tier 1 genomic applications having the most evidence to support their early detection and intervention [10,11]. However, a considerable portion of affected individuals remain underdiagnosed. For instance, approximately 330,000 patients are diagnosed with breast cancer every year in the United States, with an estimated 10% of these cases likely resulting from hereditary causes [12]. However, previous studies suggest that fewer than 10% of all *BRCA1* and *BRCA2* carriers have been identified [12,13]. Moreover, between 50% to 80% of individuals at risk have not received genetic testing, often due to not meeting the family history criteria outlined in current testing guidelines, and insurance rarely covers testing in such instances [12]. Among the approximately 35,000 breast cancer patients estimated to carry pathogenic *BRCA1/2* variants, only around 30% have been detected, highlighting significant gaps in identification and testing accessibility [12,14].

To address these gaps, population genetic screening (PGS) for CDC Tier 1 applications has increasingly been proposed as an approach for identifying affected individuals at the population level. The current model of family history or clinical criteria-based gene testing for hereditary cancer fails to reach many at-risk individuals [15]. Unlike these traditional methods, PGS screens the entire population, identifying at-risk individuals regardless of their family history or clinical criteria, thus improving early detection and disease prevention [14]. Though not currently recommended in clinical guidelines, the number of PGS programs nationally and globally is rising. The expenses for individuals undergoing PGS can vary significantly, ranging from zero cost to approximately $500 out-of-pocket [3]. Though health insurance typically does not cover PGS, PGS programs are growing nationally and globally [3,16]. Because the willingness of individuals to undergo PGS may be influenced by out-of-pocket expenses, many PGS programs have devised strategies to subsidize or fully cover these costs until insurance coverage is extended to include such genetic screenings [3,17,18]. These initiatives are crucial, as they aim to make PGS more accessible and affordable for individuals by minimizing financial barriers that might otherwise deter participation. For clinical settings, the Beneficiary Inducement Statue (BIS) in the United States prohibits health systems from offering or receiving remuneration in exchange for referrals or the generation of federal healthcare program business [19]. Thus, health systems’ ability to provide PGS at no or reduced cost to Medicaid and Medicare beneficiaries may be limited unless particular exemption criteria are met in this period of early clinical adoption [3].

Currently, patients’ willingness to pay (WTP) for PGS is largely unknown. Additionally, determining what factors, such as health insurance type, may be associated with WTP for PGS is crucial in understanding the value individuals place on this technology and its potential benefits [20]. If WTP for PGS differs by insurance type, individuals with certain forms of coverage may be less willing to pay out-of-pocket, which could result in lower uptake and contribute to more disparities in access to PGS. The objective of our study was to evaluate the public’s WTP for PGS and to assess whether this is associated with health insurance coverage, in order to better inform equity and implementation considerations as PGS expands. The findings from this study are intended to generate future hypotheses about the public’s willingness to pay for PGS and strategies to overcome potential barriers to cost.

## 2. Materials and Methods

### 2.1. Data Source

This study analyzed cross-sectional online survey data collected as part of a separate omnibus survey designed to examine health behaviors including healthcare access, health screening, nutrition and physical activity, and other health-related topics, as well as demographics. In January 2023, a national online convenience sample of 602 US adults was collected through the Qualtrics Online Panel platform [21]. The Qualtrics panel is a non-probability, opt-in online panel in which individuals voluntarily register to participate in survey research and are recruited through multiple sources, including targeted email lists, customer loyalty portals, social media, and other online platforms. Panel members are identified and invited by Qualtrics based on pre-specified eligibility criteria.

Participants were eligible if they were over the age of 18 and resided in the US at the time of the survey. The survey was a one-time, self-administered questionnaire that required approximately 15 min to complete for which participants provided informed consent electronically prior to participation. Upon completion of this survey, participants received incentives of a reward type and amount set by Qualtrics, the survey vendor (e.g., cash, reward points).

The data collected through the Qualtrics Online Panel were provided to the researchers in a de-identified format, ensuring the privacy and confidentiality of all participants. Therefore, ethical approval or written informed consent for this secondary data analysis was not required by the Office of Human Research Ethics, in accordance with the University of North Carolina at Chapel Hill Institutional Review Board (IRB) guidelines (IRB-20-2338), specifying that secondary analyses of existing data in which investigators do not have access to identifiable private information are exempt from IRB review.

### 2.2. Variables and Measurement

**Figure 1:**
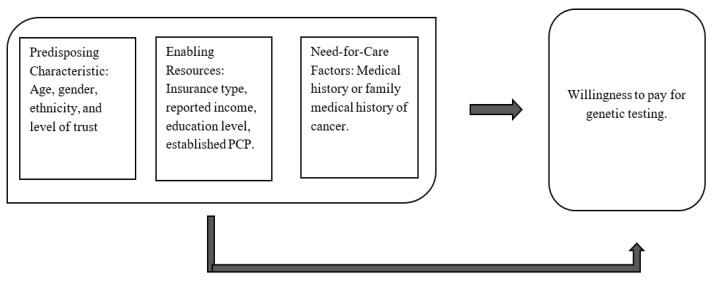
Conceptual framework based on Andersen’s behavioral model [22]. Adapted from Andersen’s behavioral model, the figure shows how predisposing characteristics, enabling resources, and need-related factors were conceptualized as influencing individuals’ stated willingness to pay for genetic testing.

This analysis was guided by Andersen’s model of health behavior and empirical evidence which posits that three sets of independent variables influence health services utilization: predisposing factors (such as demographics), enabling factors (including resources like income and insurance), and need factors (health status and perceived illness) (Figure 1) [22]. For predisposing factors, we included independent variables for age, gender, ethnicity, and level of trust towards a healthcare provider. In our enabling factors group, we included types of insurance, education level, reported income, and whether participants had an established primary care physician (PCP). Lastly, for our need factors, we included whether the participants had a personal or family medical history of cancer, since PGS included testing for certain hereditary cancers [1,2].

All variables included in our analysis were self-reported by survey respondents. Insurance type was our key independent variable and was categorized as Medicaid, Medicare, other (including Tricare, VA, IHS, or other and employer insurance), and self-pay/no insurance. Gender was measured as a categorical variable and grouped into male, female, and other. The participant’s education level was categorized as less than high school, high school graduate (or GED), some college or technical school, associate degree, bachelor’s degree, and graduate or professional degree. Ethnicity was grouped in a binary variable (Hispanic versus non-Hispanic). Level of trust was measured as high for participants reporting a high or some trust and low for participants reporting little or no trust in the health care system. Reported household income was grouped into the following categories: $0–$24,999, $25,000–$49,999, $50,000–$74,999, $75,000–$99,999, and $100,000 or more. Whether patients had an established PCP or not was measured as a binary variable. Past medical history of cancer, which included those with either a current or past diagnosis of cancer, and family medical history of cancer were created as binary variables. Age was measured in chronological years as a continuous variable. We also included age as a quadratic variable because it can help capture the possibility that the effect of age on WTP for PGS increases (or decreases) at an accelerating rate as people get older.

Our dependent variable was willingness to pay for population genetic screening measured as an ordered categorical variable identifying participants’ willingness to pay for a genetic test.

Patients were randomized to receive differing costs in the question stem referring to WTP ($0, $100, $250, or $500). The question presented to participants was: ‘What is the maximum amount you would be willing to pay for a genetic test that analyzes your DNA for potential cancer and cardiovascular risks?’ As such, we controlled for which question stem the respondent received to account for potential anchoring. We combined response categories to match current costs of PGS across existing programs in the US [23]. The categories included $0 or none, $1–$50, $51–$100, $101–$250, $251–$500, and $501 or more.

### 2.3. Inclusion and Exclusion Criteria

We employed a complete case analysis selectively considering only those cases where complete information was available for all the relevant variables defined by our inclusion and exclusion criteria [24].

### 2.4. Data Analysis

We calculated summary statistics, including percentages, to describe the characteristics of our sample. Differences between groups were assessed using *t*-tests for continuous variables and Pearson’s chi-squared tests for categorical variables.

Then, we developed an ordered probit (oprobit) model to estimate the relationship between the WTP variable and insurance type, while controlling for the aforementioned covariables informed by Anderson’s behavioral model. An ordered probit model was used to account for the ordinal (ranked) structure of the outcome while estimating the probability of higher response categories. We estimated robust average marginal effects to assess the marginal effect of covariates on the probability of a category of WTP occurring. Statistical analyses, tables, and figures were performed using STATA software version 17.0 (StataCorp., College Station, TX, USA, 2023) and RStudio version 2023.03.1+446 (Posit Software, PBC, Boston, MA, USA, 2023) [25,26].

## 3. Results

### 3.1. Sample Statistics

**Figure 2:**
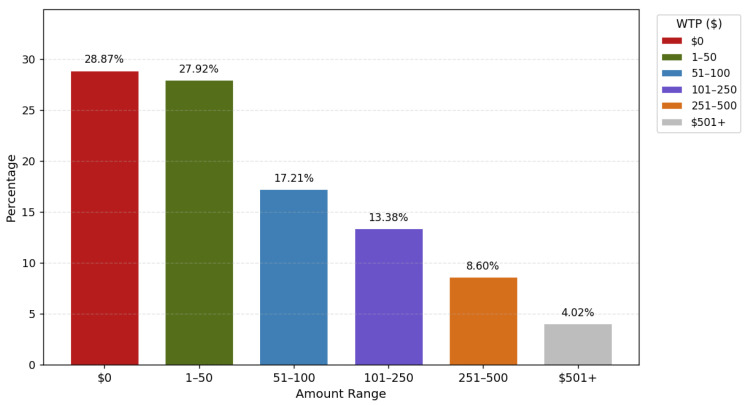
Willingness to pay (WTP) distribution across our sample. The bars in this figure represent the percentage of respondents selecting each predefined cost category ($0, $1–$50, $51–$100, $101–$250, $251–$500, and ≥$501) when asked the maximum amount they would be willing to pay (WTP) for PGS. Percentage values are displayed above each bar.

Among the 602 respondents, 524 (87%) had complete data and were included in our analysis. Characteristics of the sample are presented in Table 1. In our overall sample, most were insured by Medicare, Medicaid, or other insurance (35%, n = 181 Medicare; 27%, n = 143 Medicaid; 27%, n = 140 other insurance) and 12%, n = 60, were uninsured or self-pay. The majority of the respondents identified as women (72%, n = 378), and as being White (84%, n = 441) (Table 1). The distribution of WTP across ordered categories was right-skewed, with the highest concentration of responses in the lower payment categories ($0 and $1–$50) and progressively fewer respondents selecting higher cost thresholds (Figure 2). More than 70% of participants were willing to pay more than $0 for PGS (Figure 2).

A similar proportion of respondents with Medicare and Medicaid were willing to pay for PGS (68% and 70%, respectively), as well as with self-pay/no insurance and other (69% and 77%, respectively). The proportion of respondents willing to pay for PGS decreased as costs increased (Figure 2). For example, 28% of respondents were willing to pay $1–$50 whereas only 4% were willing to pay more than $500. We also compared included and excluded observations on available demographic characteristics and did not observe substantial differences.

### 3.2. Association Between Willingness to Pay and Insurance Type

There was no statistically significant association between type of insurance and the probability of expressing any specific level of WTP for genetic testing.

Having low levels of trust towards a healthcare provider was associated with a 6.50% increase in the probability of expressing a WTP of $0 for PGS (*p* < 0.05) and 2.18% and 1.58% decreases in the probabilities of expressing a WTP of $251–500 and $501+ for PGS (*p* < 0.05), respectively, compared to those with higher levels of trust towards a healthcare provider, holding other variables constant.

Regarding race, Black race was associated with a 10.13% decrease in the probability of expressing a WTP of $0 for PGS (*p* < 0.05) and 1.70% and 3.54% increases in the probability of expressing a WTP of $51–100 (*p* < 0.01) and $101–250 for PGS (*p* < 0.05), respectively, compared to White race, holding other variables constant. Reporting being “other race” was associated with a 1.49% increase in the probability of expressing a WTP of $51–100 (*p* < 0.05), compared to White, holding other variables constant.

Having a household income between $25,000 to $49,999 annually, compared to $0–$24,999, holding other variables constant, was associated with a 2.04% increase in the probability of expressing a WTP of $501 or more for PGS (*p* < 0.05). Similarly, individuals with annual household incomes ranging from $50,000 to $74,999 per year were associated with a 2.05% increase, while those with a household income between $75,000 to $99,999 annually were associated with a 4.20% increase in the probability of expressing a WTP of $501 or more for PGS (*p* < 0.05). Furthermore, individuals with household incomes of $100,000 or more per year were associated with a 4.81% increase in probability of expressing a WTP of $501 or more for PGS, all compared to those reporting $0–$24,999 in annual household income (*p* < 0.05). Having a higher income was positively associated with the probability of expressing WTP categories greater than $0, whereas it was negatively associated with the probability of expressing WTP of $0 for PGS, compared to those earning $0–$24,999 annually (*p* < 0.01), holding other variables constant.

Higher levels of education were positively associated with a willingness to pay (WTP) of $501 or more compared to individuals with less than a high school education (*p* < 0.01). Conversely, an inverse relationship was observed for the probability of a $0 WTP, indicating that higher education levels were associated with a lower probability of expressing a WTP of $0 (*p* < 0.01) for PGS (Table 2). Similarly, each additional year of age was positively associated with the probability of expressing a WTP of $0 for PGS (*p* < 0.05) and negatively associated with the probabilities of expressing a WTP of $101–250, $251–500, or $501+ (*p* < 0.05), holding other variables constant.

Lastly, respondents without a family history of cancer were associated with a 7.92% increase in the probability of expressing a WTP of $0 for PGS (*p* < 0.05) and decreases of 1.72%, 2.66%, 2.65%, and 1.93% in the probabilities of expressing a WTP of $51–100, $101–250, $251–500, and $501+, respectively (*p* < 0.05), compared to those with a family history of cancer, holding other variables constant.

We did not find significant associations between seeing a PCP, gender, ethnicity, or personal history of cancer and the probability of expressing any specific level of WTP for PGS.

## 4. Discussion

PGS presents a promising avenue for identifying individuals at elevated risk of specific cancers and cardiovascular conditions, fostering early intervention and personalized healthcare strategies [1,2,3,4,27,28]. Our study sought to evaluate the impact of healthcare insurance coverage on individuals’ WTP for genetic testing. Understanding this dynamic can be crucial in shaping accessibility and affordability of PGS services, especially in the context of laws such as the BIS, which may limit offering clinical PGS at no cost to Medicare and Medicaid beneficiaries [19].

In our study, the majority of participants expressed willingness to pay within the range of $1 to $250 for genetic testing. Notably, about 70% of respondents were willing to pay more than $0 for PGS, indicating a broad interest in PGS and a willingness to cover some cost. However, approximately 26% of respondents indicated a maximum WTP of over $100 for a genetic test, suggesting that many individuals may indeed be priced out of testing given current costs of PGS panels from commercial genetic testing companies [29].

Strategies to subsidize and cover the costs of PGS during its early adoption can play a significant role in reducing potential health inequities and ensuring broad accessibility to genetic testing services [3]. Drawing from the findings outlined by Washington et al. and other initiatives, various mechanisms have been proposed, including leveraging research allocations, government support, integration within healthcare systems, direct subsidies, and collaborative partnerships to offset expenses until insurance coverage expands to include PGS [3,19]. These strategies may reduce financial barriers and promote more equitable access to genetic testing as programs expand, potentially laying the groundwork for widespread adoption.

Studies have shown that having established trust in healthcare professionals can lead to better health behaviors, satisfaction, and overall health outcomes [28]. Therefore, we observed how individuals with low trust had a higher probability of being willing to pay the lowest price category (not WTP more than $0), which could be interpreted as a cautious approach to investing in healthcare services from the patients’ perspective, possibly due to skepticism about the system’s effectiveness. This aligns with the broader understanding that mistrust may drive patients to either avoid higher healthcare spending due to doubts about the benefits or to only engage minimally where the financial risk is lower [28]. This level of trust can encourage individuals to invest more in their health care, expecting better health outcomes and more personalized care. This is supported by previous research indicating that strong patient–provider relationships, marked by trust, are linked to more positive health behaviors and are the foundation of effective treatments [30,31].

When discussing racial differences in WTP for PGS, Black respondents exhibited a statistically higher WTP in the $51–100 and $101–250 price categories compared to White respondents. This could suggest that Black individuals may perceive a higher value or have a greater perceived need for genomic screenings within these specific price ranges. This finding aligns with research by White et al., which suggests a growing receptivity among African Americans to genetic explanations and research [32].

Higher household incomes were associated with an increased probability of higher WTP for PGS ($51 or more), aligning with economic theories suggesting that individuals with greater financial resources are more inclined to invest in perceived beneficial healthcare services [33]. Additionally, higher education levels (those with a high school diploma or higher education achieved) were associated to increased WTP for PGS ($51 or more), suggesting that certain educational attainments may influence the perceived value and understanding of PGS, impacting individuals’ readiness to invest in such screenings [34].

Age was positively associated with WTP at the $0 category, indicating that older individuals were more likely to report no willingness to pay for PGS. As cost thresholds increased beyond $100, the probability of selecting higher WTP categories declined with age. In contrast, prior research has observed greater willingness among younger individuals to incur out-of-pocket expenses for health innovations like genetic testing, possibly due to less fatalistic attitudes towards diseases and a higher value placed on preventive measures [34].

Our results appear consistent with previous research suggesting that individuals with a strong family history of hereditary cancer may be less WTP for genetic testing due to an assumed positive result or a familiarity with necessary preventive measures, and they provide further insight into the influence of family history on health-related financial decisions for PGS [20]. Specifically, we observe that individuals without a family history of hereditary cancer show greater willingness to pay lower amounts ($0 and $1–50), but are significantly less willing to pay higher amounts ($51 and above), supporting the notion that personal experience and familiarity with a disease can shape one’s perception of risk and the value of preventive interventions [20].

The absence of a significant association between insurance type and WTP may reflect several factors. First, limited population-level awareness of genetic tests and their coverage may constrain respondents’ ability to factor insurance into their preference statements, given documented gaps in understanding and access to genetic testing services [35]. Second, hypothetical bias inherent in contingent valuation and stated preference methods may distort WTP measures, as hypothetical willingness often differs from real payment behavior [36,37]. Finally, respondents’ perceived value of genetic information may outweigh considerations of insurance coverage when evaluating WTP [38].

Although several associations reached statistical significance, most marginal effects for higher WTP categories were in the range of 2 to 5 percentage points. These estimates indicate measurable differences in predicted probabilities, which may be meaningful when considered in the context of population-level implementation of PGS.

There was no statistically significant association between health insurance, having an established PCP, gender, ethnicity, having a history of cancer, and the probability of expressing a given WTP category.

It is important to acknowledge some limitations in our study. First, our study relied on a relatively small, convenience-based online panel with an overrepresentation of White and female respondents compared with the general US population. This imbalance likely reflects, at least in part, documented disparities in awareness and uptake of genetic testing. Previous studies utilizing nationally representative data have demonstrated that non-Hispanic White adults have significantly more knowledge of cancer genetic testing compared with other racial and ethnic groups [28], and female sex has consistently been associated with greater likelihood of genetic testing awareness and utilization [39]. Because our study required engagement with genetic testing concepts within the survey questions, individuals already aware of or interested in genetic testing may have been more likely to participate. Nevertheless, this sampling pattern limits generalizability, particularly with respect to insurance coverage, and WTP among Medicaid beneficiaries and other underserved populations. We used the Qualtrics Online Panel for national recruitment; this platform functions as a non-probability, opt-in online panel that aggregates respondents from multiple sources using a variety of recruitment methods [40]. As a result of this recruitment structure, the total number of invitations distributed, response rate, and completion rate are not disclosed to researchers. Consequently, a conventional response rate could not be calculated, and nonresponse bias could not be formally assessed. Additionally, we relied on self-reported data, potentially introducing social desirability bias, where participants may provide answers they perceived as socially acceptable. Additionally, our study had a cross-sectional design, making it difficult to establish causality. For a more comprehensive understanding of the observed trends, future research may consider employing larger sample sizes and diversifying data collection methods, such as incorporating qualitative interviews or mixed-methods approaches, as this could provide a more comprehensive understanding of the observed trends. These steps can enhance the robustness and generalizability of the findings, allowing for more precise recommendations and interventions. Another study limitation was the challenges of using a categorical data model. A continuous variable might offer a more detailed analysis of specific WTP thresholds. We were unable to discern whether individuals in the $50–100 category would be inclined to pay closer to $50 or closer to $100, so we are unable to provide as detailed recommendations about how to price or subsidize testing to ensure accessibility. It is crucial to note that we did not have data on family or personal history of high cholesterol (related to familial hypercholesterolemia, a CDC Tier 1 condition) in our survey and therefore could not control for this variable in our analysis. Furthermore, our study concentrated on health insurance types, and future investigations could explore additional contextual factors, such as geographic location, cultural beliefs, or personal attitudes towards genetic testing.

Although current market offerings for PGS might price the test at $250 or higher, our findings reveal that only around 12% of our sample showed WTP beyond the prevailing market prices and the affordability threshold perceived by individuals. These findings imply that there may be a need for alternative options to enhance the availability of PGS. One alternative approach, as discussed by Washington et al., involves research funding to support testing for such populations, potentially offering a more effective avenue to enhance access to PGS [19]. Other options such as state-funded programs or waivers from accountable care organizations could facilitate broader access to PGS. Additionally, health care reform efforts may offer another avenue to ensure equitable access to PGS and alleviate burdensome costs for patients, although this process typically involves a stringent political process and extended timelines for resolution. Future work can examine the relative impact of these strategies for improving equitable access to PGS.

## 5. Conclusions

In conclusion, WTP for PGS was not significantly associated with insurance type. Although the majority of respondents reported some level of WTP, approximately one-third expressed no WTP for PGS, and responses overall were concentrated in lower out-of-pocket cost categories. These findings suggest variability in WTP for PGS and add to the limited literature on how individuals value genomic screening tests.

## Figures and Tables

**Table 1:** Characteristics of study sample, stratified by willingness to pay for genetic testing, from Omnibus 2022.

Variable	Total (n = 524)	Willing to Pay for Genetic Testing (n = 373)	Not Willing to Pay for Genetic Testing (n = 151)	*p*-Value ^f^
**Insurance Type ^a^, n (%)**				0.431
Medicaid	143 (26.7%)	99 (26.5%)	41 (27.0%)	
Medicare	179 (34.1%)	121 (32.4%)	58 (38.2%)	
Self-pay/no insurance	61 (11.6%)	42 (11.3%)	19 (12.5%)	
Other	145 (27.6%)	111 (29.8%)	34 (22.4%)	
**Level of Trust ^b^, n (%)**				0.007 *
High	327 (62.3%)	246 (66.0%)	81 (53.3%)	
Low	198 (37.7%)	127 (34.0%)	71 (46.7%)	
**Established PCP ^c^, n (%)**				0.268
Seeing a PCP	355 (67.6%)	262 (70.2%)	93 (61.2%)	
Not seeing a PCP	170 (32.4%)	111 (29.8%)	59 (38.8%)	
**Gender, n (%)**				0.122
Woman	378 (72.0%)	264 (70.8%)	114 (75.0%)	
Man	142 (27.0%)	104 (27.9%)	38 (25.0%)	
Other	5 (1.0%)	5 (1.3%)	0 (0.0%)	
**Race, n (%)**				0.097
White	441 (84.0%)	306 (82.0%)	135 (88.8%)	
Black	47 (9.0%)	36 (9.7%)	11 (7.2%)	
Other	37 (7.0%)	31 (8.3%)	6 (3.9%)	
**Ethnicity, n (%)**				0.021 *
Hispanic	40 (7.6%)	33 (8.8%)	7 (4.6%)	
Non-Hispanic	485 (92.4%)	340 (91.2%)	145 (95.4%)	
**Level of Education, n (%)**				0.041 *
Less than high school	15 (2.9%)	6 (1.6%)	9 (5.9%)	
High school graduate	136 (26.0%)	89 (23.9%)	47 (30.9%)	
Some college	123 (23.4%)	88 (23.6%)	35 (23.0%)	
Associate degree	51 (9.7%)	39 (10.5%)	12 (7.9%)	
Bachelor’s degree	145 (27.6%)	106 (28.4%)	39 (25.7%)	
Graduate degree	55 (10.5%)	45 (12.1%)	10 (6.6%)	
**Age, mean (SD)**	53.6 (18.6)	52.6 (18.9)	56.2 (17.7)	0.127
**Household Income, n (%)**				0.786
$0–$24,999	118 (22.5%)	74 (19.8%)	44 (28.9%)	
$25,000–$49,999	145 (27.6%)	102 (27.4%)	43 (28.3%)	
$50,000–$74,999	109 (20.8%)	79 (21.2%)	30 (19.7%)	
$75,000–$99,999	72 (13.7%)	54 (14.5%)	18 (11.8%)	
$100,000 or more	81 (15.4%)	64 (17.2%)	17 (11.2%)	
**Hx of Cancer ^d^, n (%)**				0.008 *
Personal Hx	442 (84.2%)	313 (83.9%)	129 (84.9%)	
No personal Hx	83 (15.8%)	60 (16.1%)	23 (15.1%)	
**Family Hx of Cancer ^e^, n (%)**				0.044 *
Family Hx	326 (62.1%)	245 (65.7%)	81 (53.3%)	
No family Hx	199 (37.9%)	128 (34.3%)	71 (46.7%)	

^a^ Insurance coverage reported by respondents. ^b^ Level of trust towards the healthcare provider. ^c^ Established care with a primary care physician (PCP). ^d^ Personal history (Hx) of any past or present cancer diagnosis. ^e^ Family history (Hx) of any cancer. ^f^ *p*-values reported for *t*-tests of continuous variables and chi-squared tests of categorical variables. * *p* < 0.05 indicating statistical significance.

**Table 2:** Robust marginal effects for willingness to pay categories for genetic testing.

Robust Marginal Effects (n = 524)						
	WTP					
Variables	$0	$1–50	$51–100	$101–250	$251–500	$501+
**Insurance Type**						
Medicaid	ref.	ref.	ref.	ref.	ref.	ref.
Medicare	0.012	0.002	0.0024	−0.0041	−0.0043	−0.0032
Self-pay/no insurance	0.0187	0.0029	−0.0039	−0.0063	−0.0065	−0.0049
Other	0.0154	0.0025	−0.0031	−0.0052	−0.0054	−0.0041
**Level of Trust**						
High	ref.	ref.	ref.	ref.	ref.	ref.
Low	0.0650 *	0.0088	−0.0142	−0.0219	−0.0218 *	−0.0158 *
**Established PCP**						
Seeing a PCP	ref.	ref.	ref.	ref.	ref.	ref.
Not seeing a PCP	0.0435	0.0058	−0.0094	−0.0145	−0.0146	−0.0108
**Gender**						
Male	ref.	ref.	ref.	ref.	ref.	ref.
Female	0.0433	0.0076	−0.0087	−0.0148	−0.0155	−0.0118
Other	−0.1346	−0.0647	0.0064	0.0454	0.0692	0.0782
**Race**						
White	ref.	ref.	ref.	ref.	ref.	ref.
Black	−0.1013 *	−0.0264	0.0170 **	0.0354 *	0.0408	0.0343
Other	−0.0819	−0.0188	0.0149 *	0.0286	0.0316	0.0256
**Ethnicity**						
Hispanic	ref.	ref.	ref.	ref.	ref.	ref.
Non-Hispanic	0.0499	0.0107	−0.0093	−0.0172	−0.0188	−0.0151
**Level of Education**						
Less than high school	ref.	ref.	ref.	ref.	ref.	ref.
High school graduate	−0.2166 *	0.0332	0.0603 *	0.0582 *	0.0427 *	0.0220 *
Some college	−0.2815 *	0.0258	0.0751 *	0.0800 **	0.0637 **	0.0367 **
Associate degree	−0.3129 *	0.0183	0.0809 *	0.0910 **	0.0759 **	0.0466 *
Bachelor’s degree	−0.2786 *	0.0264	0.0745 *	0.0790 **	0.0626 **	0.0359 **
Graduate degree	−0.3247 *	0.0147	0.0827 *	0.0953 **	0.0809 **	0.0509 **
**Age**						
Age (years)	0.0029 *	0.0008 *	−0.00046	−0.00102 *	−0.0012 *	−0.0011 *
**Household Income**						
$0–$24,999	ref.	ref.	ref.	ref.	ref.	ref.
$25,000–$49,999	−0.1181 *	−0.0029	0.0299 *	0.0376 *	0.0329 *	0.0204 *
$50,000–$74,999	−0.1180 *	−0.0029	0.0299 *	0.0376 *	0.0329 *	0.0205 *
$75,000–$99,999	−0.1855 **	−0.0201	0.0421 **	0.0617 **	0.0597 **	0.0420 *
$100,000 or more	−0.1995 **	−0.0255	0.0439 **	0.0668 **	0.0662 **	0.0481 *
**History of Cancer**						
Personal history	ref.	ref.	ref.	ref.	ref.	ref.
No personal history	0.003	0.0004	−0.0006	−0.00103	−0.00106	−0.0008
**Family History of Cancer**						
Family history	ref.	ref.	ref.	ref.	ref.	ref.
No family history	0.0792 *	0.01044 *	−0.0172 *	−0.02657 *	−0.02653 *	−0.01928 *

Acronyms: primary care physician (PCP), history (Hx). Table includes coefficients and standard errors in parenthesis. “Ref.” represents the references categories in our model. * *p* < 0.05 ** *p*< 0.001.

## Data Availability

The data presented in this study are available on request from the corresponding author, as data that support the findings of this study are not publicly available as they are secondary data collected from another study with a small sample size.

## Abbreviations

The following abbreviations are used in this manuscript:

PGS	Population genetic screening
WTP	Willingness to pay
BIS	Beneficiary Inducement Statute
*BRCA1*	Breast cancer susceptibility gene 1
*BRCA2*	Breast cancer susceptibility gene 2
CDC	Centers for Disease Control and Prevention
PCP	Primary care physician

## References

[1] Roberts M.C., Foss K.S., Henderson G.E., Powell S.N., Saylor K.W., Weck K.E., Milko L.V. (2022). Public Interest in Population Genetic Screening for Cancer Risk. Front. Genet..

[2] Shen E.C., Srinivasan S., Passero L.E., Allen C.G., Dixon M., Foss K., Halliburton B., Milko L.V., Smit A.K., Carlson R. (2022). Barriers and Facilitators for Population Genetic Screening in Healthy Populations: A Systematic Review. Front. Genet..

[3] Foss K.S., O’daniel J.M., Berg J.S., Powell S.N., Cadigan R.J., Kuczynski K.J., Milko L.V., Saylor K.W., Roberts M., Weck K. (2022). The Rise of Population Genomic Screening: Characteristics of Current Programs and the Need for Evidence Regarding Optimal Implementation. J. Pers. Med..

[4] Manchanda R., Sideris M. (2022). Population-based genetic testing for cancer susceptibility genes: Quo vadis?. BJOG Int. J. Obstet. Gynaecol..

[5] Lee C.-L., Chuang C.-K., Chiu H.-C., Chang Y.-H., Tu Y.-R., Lo Y.-T., Lin H.-Y., Lin S.-P. (2025). Understanding Genetic Screening: Harnessing Health Information to Prevent Disease Risks. Int. J. Med. Sci..

[6] Das S., Dey M.K., Devireddy R., Gartia M.R. (2023). Biomarkers in Cancer Detection, Diagnosis, and Prognosis. Sensors.

[7] Manchanda R., Patel S., Gordeev V.S., Antoniou A.C., Smith S., Lee A., Hopper J.L., MacInnis R.J., Turnbull C., Ramus S.J. (2018). Cost-effectiveness of Population-Based BRCA1, BRCA2, RAD51C, RAD51D, BRIP1, PALB2 Mutation Testing in Unselected General Population Women. JNCI J. Natl. Cancer Inst..

[8] National Academies of Sciences, Engineering, and Medicine (2023). Using Population Descriptors in Genetics and Genomics Research: A New Framework for an Evolving Field.

[9] Patel A.P., Wang M., Fahed A.C., Mason-Suares H., Brockman D., Pelletier R., Amr S., Machini K., Hawley M., Witkowski L. (2020). Association of Rare Pathogenic DNA Variants for Familial Hypercholesterolemia, Hereditary Breast and Ovarian Cancer Syndrome, and Lynch Syndrome With Disease Risk in Adults According to Family History. JAMA Netw. Open.

[10] Dotson W.D., Kolor K., Khoury M.J., Grosse S.D. (2021). Tier 1 Guidelines on Family-Based Screening for Hereditary Hemochromatosis. Genomics and Precision Health Blog.

[11] Centers for Disease Control and Prevention (2014). Tier 1 Genomics Applications and their Importance to Public Health. CDC Archive.

[12] Beitsch P.D., Whitworth P.W., Hughes K., Patel R., Rosen B., Compagnoni G., Baron P., Simmons R., Smith L.A., Grady I. (2019). Underdiagnosis of Hereditary Breast Cancer: Are Genetic Testing Guidelines a Tool or an Obstacle?. J. Clin. Oncol..

[13] Pederson H.J., Narod S.A. (2024). Commentary: Why is genetic testing underutilized worldwide? The case for hereditary breast cancer. BJC Rep..

[14] McAlarnen L., Stearns K., Uyar D. (2021). Challenges of Genomic Testing for Hereditary Breast and Ovarian Cancers. Appl. Clin. Genet..

[15] Zhang L., Bao Y., Riaz M., Tiller J., Liew D., Zhuang X., Amor D.J., Huq A., Petelin L., Nelson M. (2019). Population genomic screening of all young adults in a health-care system: A cost-effectiveness analysis. Genet. Med..

[16] Garavito G.A.A., Moniz T., Déom N., Redin F., Pichini A., Vindrola-Padros C. (2022). The implementation of large-scale genomic screening or diagnostic programmes: A rapid evidence review. Eur. J. Hum. Genet..

[17] National Academies of Sciences, Engineering, and Medicine (2018). Financial Considerations for Implementing Genomics-Based Screening Programs. Implementing and Evaluating Genomic Screening Programs in Health Care Systems: Proceedings of a Workshop.

[18] Ries N.M., Hyde-Lay R., Caulfield T. (2009). Willingness to pay for genetic testing: A study of attitudes in a Canadian population. Public Health Genom..

[19] Washington A.M., Foss K., Krause J.H., Davis A.M., Kuczynski K.J., Milko L.V., Berg J.S., Roberts M.C. (2023). Consideration of the Beneficiary Inducement Statute on Access to Health Care Systems’ Population Genetic Screening Programs. Public Health Genom..

[20] Matro J.M., Ruth K.J., Wong Y., McCully K.C., Rybak C.M., Meropol N.J., Hall M.J. (2014). Cost sharing and hereditary cancer risk: Predictors of willingness-to-pay for genetic testing. J. Genet. Couns..

[21] Qualtrics (2022). Online Survey Software & Insight Platform.

[22] Andersen R.M. (1995). Revisiting the behavioral model and access to medical care: Does it matter?. J. Health Soc. Behav..

[23] Foss K., Henderson G., Milko L., O’Daniel J., Garner D., Roberts M.C. Learning from each other: Understanding strategies to increase the reach and representativeness of population genetic screening. Proceedings of the 16th Annual Conference on the Science of Dissemination and Implementation.

[24] Jamshidian M., Mata M., Lee S.-Y. (2007). 2—Advances in Analysis of Mean and Covariance Structure when Data are Incomplete**This research was supported in part by the National Science Foundation Grant DMS-0437258. Handbook of Latent Variable and Related Models.

[25] R-Core-Team (2018). R: A Language and Environment for Statistical Computing.

[26] StataCorp (2023). Stata Statistical Software: Release 18.

[27] Abul-Husn N.S., Soper E.R., Braganza G.T., Rodriguez J.E., Zeid N., Cullina S., Bobo D., Moscati A., Merkelson A., Loos R.J.F. (2021). Implementing genomic screening in diverse populations. Genome Med..

[28] Hong Y.-R., Yadav S., Wang R., Vadaparampil S., Bian J., George T.J., Braithwaite D. (2023). Genetic Testing for Cancer Risk and Perceived Importance of Genetic Information Among US Population by Race and Ethnicity: A Cross-sectional Study. J. Racial Ethn. Health Disparities.

[29] Invitae C. (2024). About Our Tests.

[30] Birkhäuer J., Gaab J., Kossowsky J., Hasler S., Krummenacher P., Werner C., Gerger H. (2017). Trust in the health care professional and health outcome: A meta-analysis. PLoS ONE.

[31] Fuehrer S., Weil A., Osterberg L.G., Zulman D.M., Meunier M.R., Schwartz R. (2024). Building Authentic Connection in the Patient-Physician Relationship. J. Prim. Care Community Health.

[32] White D.B., Koehly L.M., Omogbehin A., McBride C.M. (2010). African Americans’ responses to genetic explanations of lung cancer disparities and their willingness to participate in clinical genetics research. Anesthesia Analg..

[33] Engelgau M.M., Zhang P., Jan S., Mahal A. (2019). Economic Dimensions of Health Inequities: The Role of Implementation Research. Ethn. Dis..

[34] Aizuddin A.N., Ramdzan A.R., Omar S.A.S., Mahmud Z., Latiff Z.A., Amat S., Teik K.W., Siew C.G., Rais H., Aljunid S.M. (2021). Genetic Testing for Cancer Risk: Is the Community Willing to Pay for It?. Int. J. Environ. Res. Public Health.

[35] Dusic E.J., Theoryn T., Wang C., Swisher E.M., Bowen D.J., EDGE Study Team (2022). Barriers, interventions, and recommendations: Improving the genetic testing landscape. Front. Digit. Health.

[36] Ebert J., Winzer P., Müller C. (2025). Reducing the Hypothetical Bias in Measuring Willingness to Pay for Mobile Communication Products. J. Theor. Appl. Electron. Commer. Res..

[37] Hofstetter R., Miller K.M., Krohmer H., Zhang Z.J. (2021). A de-biased direct question approach to measuring consumers’ willingness to pay. Int. J. Res. Mark..

[38] Morrish N., Snowsill T., Dodman S., Medina-Lara A. (2024). Preferences for Genetic Testing to Predict the Risk of Developing Hereditary Cancer: A Systematic Review of Discrete Choice Experiments. Med. Decis. Mak..

[39] Makhnoon S., Maki K.G., Yu R., Peterson S.K., Shete S. (2022). Are beliefs about the importance of genetics for cancer prevention and early detection associated with high risk cancer genetic testing in the U.S. Population?. Prev. Med. Rep..

[40] Douglas B.D., Ewell P.J., Brauer M. (2023). Data quality in online human-subjects research: Comparisons between MTurk, Prolific, CloudResearch, Qualtrics, and SONA. PLoS ONE.

